# Providing Brief Personalized Therapies for Insomnia Among Workers Using a Sleep Prompt App: Randomized Controlled Trial

**DOI:** 10.2196/36862

**Published:** 2022-07-25

**Authors:** Tomonari Shimamoto, Ryuji Furihata, Yukako Nakagami, Yukiko Tateyama, Daisuke Kobayashi, Kosuke Kiyohara, Taku Iwami

**Affiliations:** 1 Department of Preventive Services, School of Public Health, Graduate School of Medicine Kyoto University Kyoto Japan; 2 Agency for Health, Safety and Environment Kyoto University Kyoto Japan; 3 Department of Food Science Faculty of Home Economics Otsuma Women’s University Tokyo Japan

**Keywords:** sleep prompt app, smartphone, brief personalized therapies for insomnia, worker, randomized controlled trial, Japan

## Abstract

**Background:**

Insomnia is the most common sleep disorder and the foremost health concern among workers. We developed a new sleep prompt app (SPA) for smartphones to positively alter the users' consciousness and behavior by sending timely short messages for mild sleep problems at an early stage.

**Objective:**

The aim of this study is to investigate the effectiveness of the SPA in providing brief personalized therapy for insomnia among workers.

**Methods:**

We conducted a 2-arm parallel randomized controlled trial. The intervention group used the SPA, and the control group received no intervention. Participants were recruited between November 2020 and January 2021. The researcher sent emails for recruitment to more than 3000 workers of 2 companies and 1 university in Japan. The SPA provided personalized prompt messages, sleep diaries, sleep hygiene education, stimulus control therapy, and sleep restriction therapy. The prompt messages were sent automatically to the participants to encourage them to improve their sleep habits and sleep status and were optimized to the individual's daily rhythm. The intervention program duration was 4 weeks. The primary outcome was a change in the Insomnia Severity Index (ISI) for the study period. The ISI was obtained weekly using a web questionnaire.

**Results:**

A total of 116 Japanese workers (intervention group n=60, control group n=56) with sleep disorders were recruited. Two participants in the intervention group were excluded from the analyses because of challenges in installing the SPA. The mean ISI scores at baseline were 9.2 for both groups; however, after 4 weeks, the mean ISI scores declined to 6.8 and 8.0 for the intervention and control groups, respectively. Primary analysis using a linear mixed model showed a significant improvement in the temporal trends of the ISI in the SPA group and in the total population (*P*=.03). Subgroup analyses of ISI-8-insomniacs revealed a significant improvement in the temporal trends of ISI in the SPA group (*P*=.01), and the CFS score for physical condition significantly improved following the intervention (*P*=.02).

**Conclusions:**

This study demonstrates the effectiveness of the SPA in providing brief personalized therapy for insomnia among Japanese workers with mild insomnia. The physical fatigue score significantly improved in ISI-8-insomniacs. Thus, SPA could play an important role in reducing the adverse effects of sleep disorders in workers. To promote the wide use of the SPA in the future, further studies are required to examine its effectiveness in other age groups and individuals with health problems.

**Trial Registration:**

University Medical Information Network Clinical Trials Registry (UMIN-CTR) UMIN000042263; https://center6.umin.ac.jp/cgi-open-bin/ctr_e/ctr_view.cgi?recptno=R000046295

## Introduction

Insomnia is the most common sleep disorder, and previous epidemiological studies revealed that almost 20% of the general adult population exhibits symptoms of insomnia [1,2]. Insomnia is associated with increased risks of mental disorders such as depression, anxiety, alcohol abuse, and psychosis [3] and physical diseases including hypertension, hyperglycemia, hyperlipidemia, obesity, and cardio-cerebrovascular events [4,5]. In workers, insomnia is considered an important problem because it is associated with reduced work performance, processing errors, accidents at work, absenteeism, reduced quality of life, and symptoms of depression even if the insomnia is mild [6]. Thus, insomnia has become one of the foremost health concerns among workers.

In the clinical field, cognitive behavioral therapy for insomnia (CBT-I) or brief therapy for insomnia (BTI), which combine multiple treatment techniques, has been shown to be effective in randomized control trials (RCTs), and these therapies are highly recommended (strong for CBT-I, conditional for BTI) for the treatment of insomnia in the US guidelines [7]. Moreover, the development of CBT-I or BTI using internet and smartphone applications has progressed, and they have been found to be as effective as treatments provided by trained clinicians [8-10]. Therefore, CBT-I or BTI using internet and smartphone applications is expected to be widely used in the health care field through further technological innovation. However, due to the use of standardized educational programs, it has been difficult to provide individualized programs, and sometimes other media or telephone support has been needed to address individual problems [11]. Furthermore, the effectiveness of the program in the health care field for mild cases has not been fully examined.

We developed a new technology to change the users' consciousness and behavior in a desirable direction by sending short messages named “prompt messages” that encourage behavioral change in line with the users' daily rhythms in using mobile devices (eg, smartphones) and by promoting awareness of lifestyle improvement. If we can detect individual sleep problems (insufficient sleep time, excessive daytime sleepiness, disturbance of sleep-wake rhythms, issues requiring improvement in sleep hygiene, a discrepancy between total time in bed and total sleep time, and reduced sleep efficiency) and use prompting technology to provide notifications that lead to sleep improvement in real-time according to an individual's daily rhythm as if a sleep specialist were present when needed, we can provide highly individualized and efficient interventions for improved sleep behavior. Hence, to provide real-time interventions, we developed a new sleep prompt app (SPA) for smartphones through industry-academia collaboration. The SPA is expected to enable early detection and intervention in mild sleep disorders.

The primary aim of this study is to investigate whether SPA can aid in improving insomnia among Japanese workers by providing brief personalized therapy.

## Methods

### Recruitment

The study was designed as a 2-arm (intervention using the SPA vs wait-list control) parallel RCT. Participants were recruited between November 2020 and January 2021. All follow-up was completed by March 2021. An email for recruitment was sent to more than 3000 workers of 2 companies and 1 university in Japan. The institutional affiliations (Kyoto University; HealthTech Laboratory, Inc; and OKI Electric Industry Co, Ltd) were displayed in the emails as the organizations conducting the study. To avoid the risk of divulging information to competing research institutions, we selected companies and universities affiliated to our research group for recruitment of participants. However, we excluded workers of departments involved in the study. Both eligible employees and their acquaintances were allowed to participate in the study. Written informed consent was obtained through participation in face-to-face information sessions or online information sessions using a web conference system. After the information sessions, all subsequent communications were conducted via email.

The inclusion criteria were workers aged 20 years or older, ownership of a smartphone, and an affirmative answer to the question “Do you have any sleep problems?” that was asked during the application for participation in this study.

There were 6 exclusion criteria: (1) individuals whose version or model of smartphone did not support the SPA, (2) shift workers, (3) individuals likely to be subjected to significant time zone changes such as overseas travel during the study period, (4) those who had a total score of 10 or more on the Patient Health Questionnaire-9 at the time of application for participation in the study, (5) those who responded that the frequency of “thoughts that you would be better off dead or of hurting yourself in some way” was more than half of the days in the previous 2 weeks, and (6) those who were judged to be inappropriate for the study.

### Randomization

Random assignment into the SPA group or nonintervention control group was performed by stratified block allocation with an allocation ratio of 1:1, a block size of 4 using sex and 3 groups of total Insomnia Severity Index (ISI) scores obtained at the time of study entry: <8, 8-14, and ≥15. Central randomization with computer-generated tables was used for the allocation. One investigator (TS) prepared the list, and another investigator (KK) independently performed the allocation. Due to the nature of the intervention, the participants were not blinded to their allocation. The researchers included statisticians (KK), and data analysts (RF and YN) who were blinded to the assignment of the participants to their respective groups.

### Interventions

#### Overview

The SPA consisted of a smartphone app (HealthTech Laboratory, Inc) and a prompt notification server (OKI Electric Industry Co, Ltd). For the intervention, the smartphone app, Kenko-Nikki (Health Diary), which is a personal health record–related app developed by HealthTech Laboratory, Inc, a Kyoto University–originated venture company in the Kyoto University Incubation Program [12], was customized for this study. Following the manufacturer's instructions, participants in the intervention group installed the app (TestFlight for iOS or DeployGate for Android) to use the SPA for free. The brief personalized therapy for insomnia used in the intervention consisted of 5 components: (1) prompt messages, (2) a sleep diary, (3) sleep hygiene education, (4) stimulus control therapy, and (5) sleep restriction therapy.

Each study lasted 28 days, and we conducted 7 trials between November 2020 and February 2021. The intensity of use or dosage for the SPA was not recorded in the research data. The participants in the control group received the SPA after completing the posttest questionnaire. During the study period, an email contact was provided for inquiries.

#### Prompt Messages

A sleep medicine specialist (RF) created more than 200 types of prompt messages, the timing of the message sending (after waking, around noon, in the evening, before bedtime), and the prompt messages’ sending conditions according to sleep habits and sleep states. Prompt messages and their transmission conditions were programmed and set up on the prompt notification server at OKI Electric Industry Co, Ltd. At the beginning of the study, information on sex, age, ISI, sleep habits, and list of times when each participant would be more (or less) receptive to prompt messages obtained from the online questionnaire was entered into the prompt notification server; data from the sleep diary, the ISI scores from weekly questionnaires, and sleep schedules set by the participant were also entered. Based on these participant data, the prompt notification server selected an appropriate prompt message and time at which participants would be receptive to the information and then automatically sent the prompt message to the participant at the optimal time. The dispatched prompt messages were received by the participants’ SPA (Figure S1, Multimedia Appendix 1).

**Figure 1:**
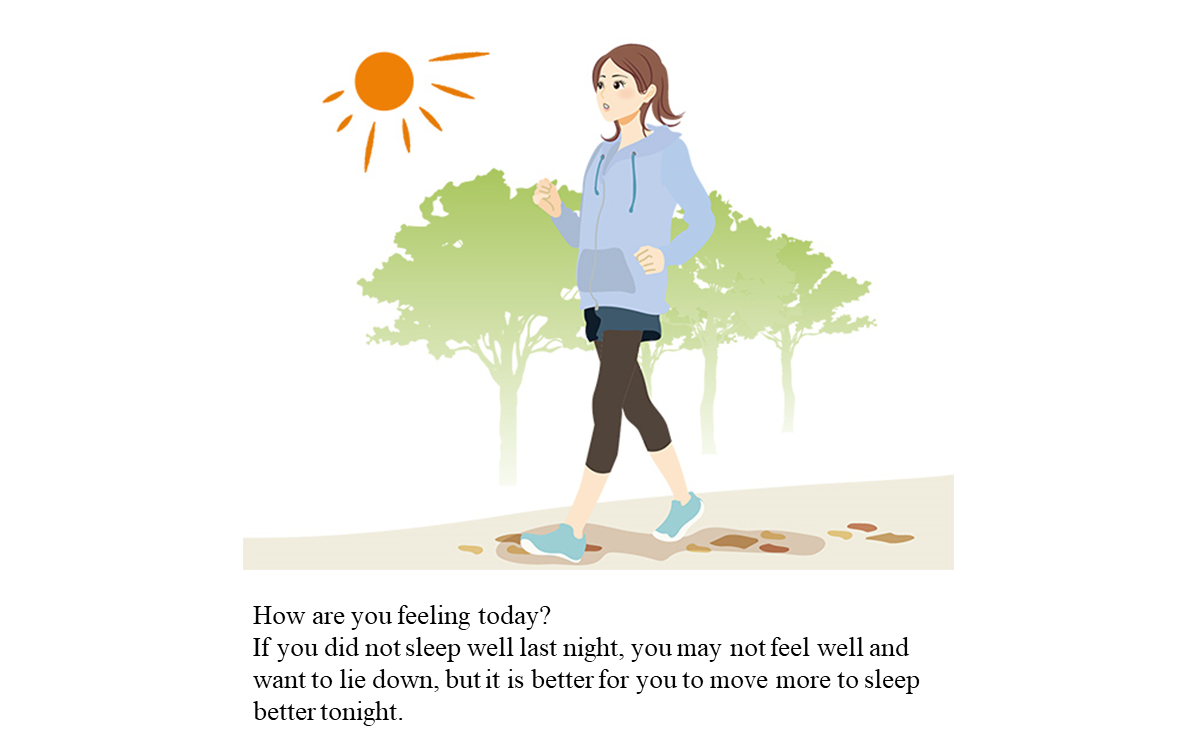
Sleep prompt app prompt message.

The prompt messages were sent with illustrations of female characters according to their content. The prompt messages consisted of comments related to sleep diaries, sleep hygiene education, stimulus control therapy, or sleep restriction therapy and were sent several times in a day on an everyday basis. An example of a prompt message is shown in Figure 1.

#### Sleep Diary Measures

**Figure 2:**
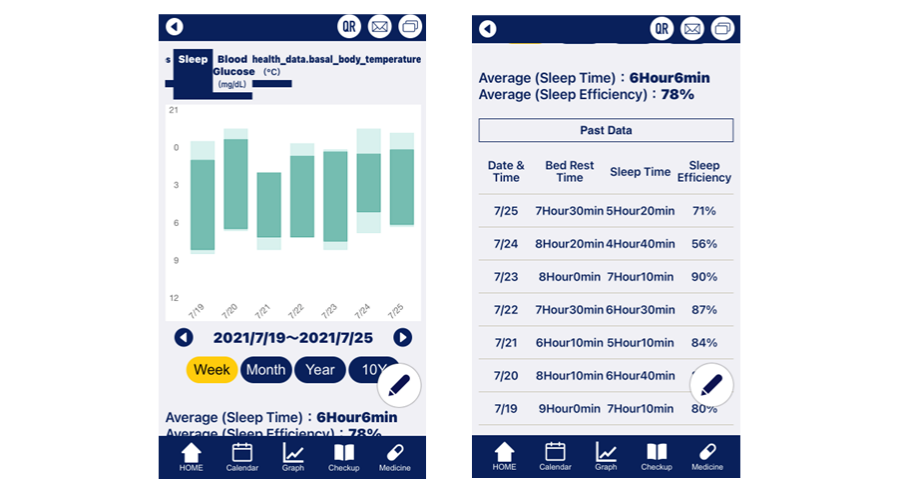
Screenshot of the sleep diary in the sleep prompt app.

The participants filled out a sleep diary daily using the SPA. In the sleep diary, they recorded the following 10 items: (1) the time of getting into bed; (2) sleep-onset latency; (3) number of awakenings; (4) duration of awakenings; (5) time of final awakening; (6) bed-out latency; (7) duration of napping; (8) time of day of feeling excessive sleepiness during daytime; (9) behavior before bedtime, including alcohol consumption, caffeine intake, smoking, and hypnotic medication use; and (10) whether the recording date was a weekday or a holiday. From these variables, the SPA calculated the time in bed (time in bed = final arising time – time of going to bed), sleep time (total sleep time = time in bed – sleep-onset latency – wake after sleep onset – bed-out latency), and sleep efficiency (sleep efficiency = [total sleep time/time in bed] × 100). In addition, the SPA presented these sleep variables in numerical and graphical forms (Figure 2). When the participants consistently entered data in their sleep diary, they received prompt messages commending them.

#### Sleep Hygiene Education and Stimulus Control Therapy

Prompt messages were sent for helpful advice on lifestyle changes related to daily rhythm, light exposure, sound, temperature, diet intake, exercise, bathing, drinking, smoking, caffeine consumption, sleep duration, napping, and emotional distress. Prompt messages of sleep hygiene instructions were sent from week 1 to 4. Recommended sleep-related behaviors based on the stimulus control method were delivered as prompt messages at weeks 2 to 4.

#### Sleep Restriction Therapy

At the end of the week, the participants set their sleep schedules for the next week. First, they entered their desired wake-up times on the SPA. The SPA presented the recommended bedtime, which was calculated by subtracting the previous week's “average total sleep time + 30 minutes” from the desired wake-up time. The participants set their desired bedtime according to the recommended bedtime. The recorded sleep schedules in the sleep diary were entered into the prompt notification server, and based on this information, the prompt notification server sent a prompt message containing advice on actual wake-up time, bedtime, and sleep efficiency. When a participant’s sleep efficiency was >90%, the SPA allowed 15 minutes extra time in bed. When a participant’s average sleep efficiency was 85%-90%, the SPA suggested the same. When a participant’s average sleep efficiency was <85%, the SPA suggested a further restriction of 15 minutes. The sleep restriction method was implemented in weeks 2-4.

### Measurements From the Online Questionnaire

All online questionnaires were distributed using Google Forms. The email which included information of the questionnaire and the URL of the questionnaire were sent to all participants.

### Primary Measure

We administered the Japanese version of the ISI questionnaire [13,14]. This is a 7-item questionnaire with scores ranging from 0 (no insomnia) to 28 (severe insomnia). Individuals with scores ≥8 were defined as ISI-8-insomniacs [13].

### Secondary Measures

#### Chalder Fatigue Scale

The Chalder fatigue scale (CFS) is a self-administered questionnaire for measuring the extent and severity of fatigue [15]. The items are benign and nonthreatening, asking about sensations and functionality—such as “Do you have problems starting things?” and “Do you have difficulty concentrating?”—rather than about any beliefs or opinions about health status. Each of the 11 items is answered on a 4-point scale ranging from asymptomatic to maximum symptomologies, such as “better than usual,” “no worse than usual,” “worse than usual,” and “much worse than usual.” According to Likert scoring, responses on the extreme left received a score of 0, increasing to 1, 2, or 3 as the participants became more symptomatic. The respondents’ global scores ranged from 0 to 33. The global score also includes 2 dimensions: physical fatigue (measured by items 1-7) and psychological fatigue (measured by items 8-11).

#### The World Health Organization Health and Work Performance Questionnaire Short Form

Work performance was assessed using the World Health Organization Health and Work Performance Questionnaire (HPQ) short form [16,17]. The HPQ includes a self-reporting assessment of the on-the-job work performance of most workers in the same job (B9), actual performance within 1 or 2 years before the survey (B10), and actual performance within 4 weeks (28 days) before the survey (B11). The on-the-job work performance scale is a 0-10 self-anchoring scale in which 0 indicates the worst performance at the workplace and 10 represents the performance of a top employee. Although several methods have been created to assess work performance, we evaluated relative presenteeism and absolute presenteeism [17]. We calculated relative presenteeism by a ratio of actual performance (B11) to the performance of most workers at the same job (B9, possible performance). Regarding the distribution of relative presenteeism, 0.25 is the worst relative performance (25% or less of other workers’ performance) and 2.0 is the best performance (200% or more of other workers’ performance) [17]. We calculated absolute presenteeism as follows: 10 × actual performance within 1 or 2 years before the survey (B11) [17].

### Other Measures

The online questionnaire included questions regarding sociodemographic information (sex and age), height, weight, caffeine consumption (calculated per day as 95 mg per cup of coffee, 55 mg per cup of tea, and 45 mg per cup of cola), alcohol consumption (never, sometimes, or every day), smoking habits (yes or no), living with others or alone, hypnotic medication (more than once a week or not), and use of alcohol as an aid to sleep (more than once a week or not). BMI was calculated using height and weight, with 2 groups being created using a cutoff of 25: obese (BMI≥25 kg/m^2^) or normal.

### Statistical Analysis

Sample-size calculations were based on our pilot study (not published) using a prototype app; the intervention produced a 1.3-point reduction (SD 2.3) on the ISI. We needed 50 participants per intervention group (100 in total) to detect 80% power using a 2-sided α of .05. Accounting for 20% potential attrition, we estimated the need to enroll 120 participants.

Descriptive statistics were computed for participant characteristics. We computed the differences in clinical and demographic variables between the intervention and control groups by original assigned groups using the independent samples *t* test for continuous variables and chi-square tests for categorical variables. As a primary analysis, linear mixed-effects models were applied to longitudinal data to measure the effectiveness of the intervention, to account for repeated measurements over time, and to avoid the imputation of missing data. These models included the ISI scores of the 2 groups, 5 time points (baseline, and 1, 2, 3, and 4 weeks later), and the time × intervention interaction. As secondary measures, we assessed the changes in absolute presenteeism score, relative presenteeism score, CFS total 11, CFS mental 11, and CFS physical 11 with an independent samples *t* test. A subgroup analysis was conducted for ISI-8-insomniacs. No interim analysis was conducted. Statistical significance was defined as *P*<.05 using 2-tailed tests. All analyses were performed using SPSS (version 27.0, IBM Corp) for Windows (Microsoft Corp).

### Ethical Considerations

The study protocol was approved by the Ethics Committee of Kyoto University (#C1478). The study was conducted in accordance with the Declaration of Helsinki. After identification at the information session and provision of written informed consent forms under real names, a correspondence chart between IDs and real names was created, and the correspondence chart was kept in strict confidence at the research department of Kyoto University. All data collected from participants' responses to the web questionnaire and the app were managed in a manner that was linked to their IDs, thus guaranteeing participants' privacy.

## Results

**Figure 3:**
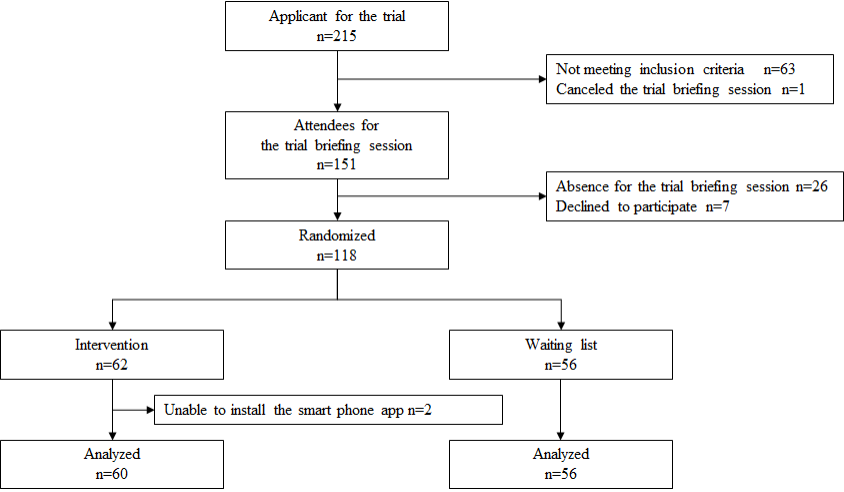
The CONSORT (Consolidated Standards of Reporting Trial) flow diagram of recruitment.

The CONSORT (Consolidated Standards of Reporting Trials) flow diagram of participant recruitment is shown in Figure 3. Of the 215 participants who participated in the study, 151 were eligible to participate in the trial briefing session, with 63 being excluded for not meeting the inclusion criteria. People with severe complications (late pregnancy and an individual with hypersomnia) and 1 who canceled participation in the trial briefing session were excluded. A further 26 were excluded because they could not attend the trial briefing session, and 7 refused to participate. Hence, 118 were eligible for allocation: 62 were assigned to the intervention group and 56 to the control group. Of the 62 participants assigned to the intervention group, 2 participants who failed to install the app were excluded from the analysis as dropouts. Finally, 116 participants were included in the analysis. No adverse effects were observed in either group. Although the number of participants did not reach 120, recruitment was terminated because the dropout rate was below the estimated number.

The participants’ characteristics (total population and ISI-8-insomniacs) are shown in Table 1. In the total population, the percentage of alcohol use as a sleep aid was significantly different between the 2 groups (*χ*^2^=4.22; *P*=.04). In ISI-8-insomniacs, the percentage of alcohol use (*χ*^2^_2_ =6.81; *P*=.03), current smoking (*χ*^2^_1_=3.90; *P*=.05), and alcohol use as a sleep aid (*χ*^2^_1_=5.85; *P*=.02) was significantly different between the 2 groups.

**Figure 4:**
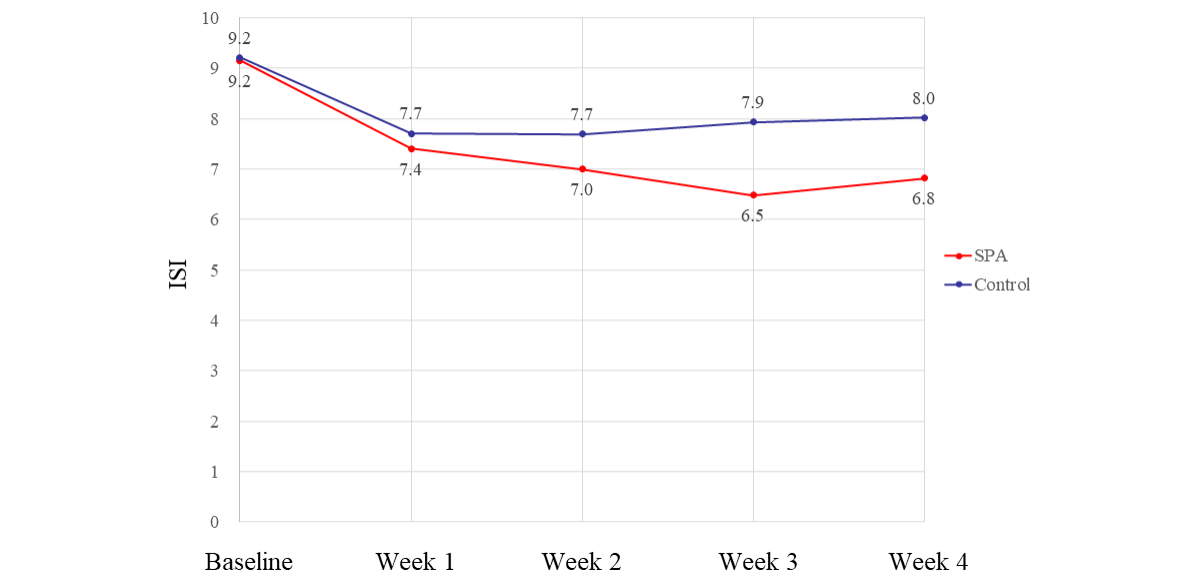
Trend in the ISI score during the 4-week trial period in the total population. ISI: Insomnia Severity Index; SPA: sleep prompt app.

Figure 4 shows the ISI scores at 4 weeks for the total population. The mean ISI scores for the SPA and control groups were 9.2 and 9.2 at baseline, 7.4 and 7.7 at 1 week, 7.0 and 7.7 at 2 weeks, 6.5 and 7.9 at 3 weeks, and 6.8 and 8.0 at 4 weeks, respectively. Statistical analysis using a linear mixed model showed that the difference in the mean ISI scores of both groups was significantly different (*P*=.03). The mean difference in the change in ISI scores of the 2 groups at baseline and after 4 weeks was 1.14 points (95% CI –2.31 to 0.04).

**Figure 5:**
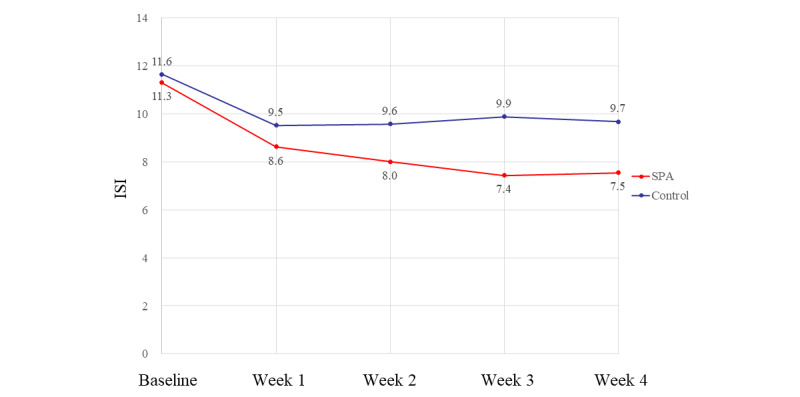
Trend in the ISI score during the 4-week trial period in the population with ISI-8-insomniacs. ISI: Insomnia Severity Index; SPA: sleep prompt app.

Figure 5 shows the ISI scores of the ISI-8-insomniacs at week 4. The mean ISI scores for the SPA and control groups were 11.3 and 11.6 at baseline, 8.6 and 9.5 at 1 week, 8.0 and 9.6 at 2 weeks, 7.4 and 9.9 at 3 weeks, and 7.5 and 9.7 at 4 weeks, respectively. Statistical analysis using a linear mixed model showed that the change in ISI scores for both groups was significantly different (*P*=.01). The mean difference in the change of ISI scores between the 2 groups at baseline and after 4 weeks was 1.79 points (95% CI –3.31 to –0.26).

The scores of presenteeism and fatigue at baseline and postintervention (total population and ISI-8-insomniacs) are shown in Table 2. In the total population, none of the variables showed a statistically significant difference. In ISI-8-insomniacs, physical CFS showed significant improvement.

The effect of SPA on sleep parameters in the sleep diary is shown in Multimedia Appendix 2 (Table S1).

**Table 1:** Participant characteristics (total population and ISI-8-insomniacs).

Variables			SPA^a^	Control
**Total population^b^**				
	Age (years), mean (SD)		38.6 (11.3)	42.7 (11.5)
	Female sex, n (%)		21 (35.0)	17 (30.4)
	Live-in alone, n (%)		18 (30.0)	13 (23.2)
	Obese (BMI ≥25 kg/m^2^), n (%)		12 (20.0)	8 (14.3)
	**Alcohol use, n (%)**			
		Never	11 (18.3)	19 (33.9)
		Sometimes	35 (58.3)	25 (44.6)
		Everyday	14 (23.3)	12 (21.4)
	Not current smoker, n (%)		55 (91.7)	54 (96.4)
	Caffeine consumption, mean (SD)		191.7 (124.1)	177.3 (138.5)
	Do not use alcohol as sleep-aid, n (%)		59 (98.3)	50 (89.3)
	Do not use hypnotic medication, n (%)		58 (96.7)	53 (94.6)
**ISI^c^-8-insomniacs^d^**				
	Age (years), mean (SD)		37.7 (11.7)	45.0 (10.7)
	Female sex, n (%)		15 (40.5)	11 (32.4)
	Live-in alone, n (%)		10 (27.0)	9 (26.5)
	Obese (BMI ≥25 kg/m^2^), n (%)		7 (18.9)	7 (20.6)
	**Alcohol use, n (%)**			
		Never	6 (16.2)	15 (44.1)
		Sometimes	23 (62.2)	13 (38.2)
		Everyday	8 (21.6)	6 (17.6)
	Not current smoker, n (%)		33 (89.2)	34 (100.0
	Caffeine consumption, mean (SD)		195.0 (137.9)	177.8 (132.7)
	Do not use alcohol as sleep-aid, n (%)		37 (100.0)	29 (85.3)
	Do not use hypnotic medication, n (%)		35 (94.6)	31(91.2)

^a^SPA: sleep prompt app.

^b^The SPA and control groups had 60 and 56 participants, respectively.

^c^ISI: Insomnia Severity Index.

^d^The SPA and control groups had 37 and 34 participants, respectively.

**Table 2:** Changes of presenteeism scores and fatigue scales before and after the intervention in the total population and ISI-8-insomniacs.

Variables			Baseline, mean (SD)	Posttest, mean (SD)	Comparison with baseline		*P* value
					Mean (SD)	95% CI	
**Total population**							
	**CFS^a^ total 11**						
		SPA^b^	15.58 (5.43)	13.35 (5.45)	–2.23 (4.35)	–2.45 to 0.87	.35
		Control	16.36 (4.77)	14.91 (5.29)	–1.45 (4.68)	N/A^c^	N/A
	**CFS mental 11**						
		SPA	6.08 (1.87)	4.45 (2.27)	–1.63 (1.75)	–0.83 to 0.52	.66
		Control	6.50 (1.88)	5.02 (2.04)	–1.48 (1.93)	N/A	N/A
	**CFS physical 11**						
		SPA	9.50 (4.11)	8.90 (3.64)	–0.60 (3.21)	–1.89 to 0.62	.32
		Control	9.86 (3.49)	9.89 (3.87)	0.04 (3.59)	N/A	N/A
	**Relative presenteeism score **						
		SPA	0.95 (0.25)	0.98 (0.21)	0.03 (0.30)	–0.08 to 0.15	.52
		Control	0.93 (0.28)	0.93 (0.23)	0.00 (0.30)	N/A	N/A
	**Absolute presenteeism score**						
		SPA	64.00 (16.59)	68.33 (15.09)	4.33 (15.44)	–4.66 to 6.90	.70
		Control	59.29 (15.36)	62.50 (15.40)	3.21 (15.97)	N/A	N/A
**ISI^d^8-insomniacs**							
	**CFS total 11**						
		SPA	17.65 (4.85)	14.38 (5.58)	–3.27 (3.73)	–4.00 to 0.11	.06
		Control	17.12 (4.57)	15.79 (5.76)	–1.32 (4.92)	N/A	N/A
	**CFS mental 11**						
		SPA	6.54 (1.77)	4.68 (2.42)	–1.86 (1.77)	–1.16 to 0.72	.64
		Control	6.74 (1.91)	5.09 (2.31)	–1.65 (2.19)	N/A	N/A
	**CFS physical 11**						
		SPA	11.11 (3.70)	9.70 (3.63)	–1.41 (2.71)	–3.21 to –0.25	.02
		Control	10.38 (3.23)	10.71 (4.10)	0.32 (3.51)	N/A	N/A
	**Relative presenteeism score **						
		SPA	0.95 (0.26)	0.98 (0.24)	0.03 (0.35)	–0.10 to 0.21	.46
		Control	0.90 (0.30)	0.87 (0.23)	–0.03 (0.31)	N/A	N/A
	**Absolute presenteeism score**						
		SPA	61.35 (16.19)	67.30 (14.07)	5.95 (12.35)	–3.77 to 10.37	.36
		Control	56.76 (15.71)	59.41 (16.69)	2.65 (17.29)	N/A	N/A

^a^CFS: Chalder fatigue scale.

^b^SPA: sleep prompt app.

^c^N/A: not applicable.

^d^ISI: Insomnia Severity Index.

## Discussion

### Principal Results

In this RCT, we examined the effectiveness of the new SPA in providing brief personalized therapy for relatively mild insomnia among Japanese workers. The results showed that the SPA significantly improved the primary outcome of ISI scores in both the total population and the ISI-8-insomniacs. In addition, the SPA also improved physical fatigue as measured by the physical CFS in ISI-8-insomniacs. To our knowledge, this is the first study to demonstrate the effectiveness of SPA in treating insomnia among workers.

### Limitations

There were several methodological limitations to our study. First, we used a subjective rating scale. The use of an objective assessment of sleep, such as polysomnography, would strengthen the results of this study. Although some studies have reported that self-reported data on sleep status concur with physiological data [18,19], using objective data (ie, physiologic measurements such as polysomnography) is desirable in future studies. Second, the participants were not blinded. The participants were aware of their assignment to the intervention or control group, and this difference could have affected the outcome. Because only those in the intervention group used the app and maintained a record in their sleep diaries, the placebo effect might have affected their behavior and influenced the results. Third, the selection of companies and universities from which participants were recruited was not random, and therefore sampling bias may exist. Fourth, the long-term prognosis of the intervention was not investigated.

### Comparison With Prior Work

Previous studies demonstrated that internet-based CBT-I improved ISI scores. One RCT compared the effectiveness of web-based CBT-I with telehealth-based CBT for chronic insomnia and found that both web- and telehealth-based treatment improved the ISI score [20]. Another RCT showed that internet-delivered CBT-I improved the ISI scores for patients with insomnia or depression [9]. In our study, the SPA group showed a statistically significant decrease in ISI score, but the difference in ISI scores between the 2 groups was smaller than that reported in previous studies [10]. In a meta-analysis of 2 previous studies, the group receiving internet-based CBT showed a decrease in the ISI score of 2.28 points (–2.89 to –1.67) compared with the control group [10]. There are some possible explanations for this. First, the low ISI scores of the study participants at baseline could have affected the results. We found a large change in the ISI scores of ISI-8-insomniacs. The fact that we were able to show the efficacy of the SPA for mild insomnia in this study is important for the establishment of future preventive methods through early interventions for sleep disorders among workers. Second, in some of the previous studies, email or phone support was required in addition to the app [9,20]. However, considering the need for widespread implementation, it is important that the SPA is effective independently, without the need of additional supportive measures.

In this study, the dropout rate was 3.2% (n=2), and most participants were able to complete the final questionnaire. In many previous studies, about 20% to 40% of the participants dropped out [11]. The app used in this study sends prompt messages to the participants at a convenient time for them, which might have contributed to maintaining their motivation and reducing the number of dropouts.

In this study, a significant improvement in physical fatigue was observed among the ISI-8-insomniacs. Fatigue symptoms have been reported as one of the most frequent daytime complaints of patients with insomnia [21], which has a major impact on day-to-day functioning and quality of life [22]. Clinical studies on patients with chronic obstructive pulmonary disease and on cancer survivors have shown that CBT-I significantly improves fatigue and insomnia in patients with insomnia [23,24]. Although no previous study has examined the effect of internet-delivered CBT-I or BTI on recovery from fatigue in workers, our intervention with the SPA showed, for the first time, the possibility of recovery from physical fatigue.

### Conclusions

This RCT showed the effectiveness of the SPA in providing brief personalized therapy for treating relatively mild insomnia among Japanese workers. The physical fatigue score was also significantly improved in ISI-8-insomniacs. The SPA could play an important role in reducing the adverse effects of sleep problems in communities. To promote the use of SPA in the future, further studies are warranted to examine its effectiveness in other age groups and individuals with health problems. To increase generalizability, future studies with follow-up surveys are required to examine the long-term prognosis of the intervention.

## Appendix

Multimedia Appendix 1 Overview of the sleep prompt app.

Multimedia Appendix 2 Effect of the sleep prompt app on the sleep parameters of the sleep diary.

Multimedia Appendix 3 CONSORT-eHEALTH checklist (V 1.6.1).

## Abbreviations

BTI: brief therapy for insomnia
CBT-I: cognitive behavioral therapy for insomnia
CFS: Chalder fatigue scale
CONSORT: Consolidated Standards of Reporting Trials
HPQ: Health and Work Performance Questionnaire
ISI: Insomnia Severity Index
RCT: randomized controlled trial
SPA: sleep prompt app
