# Supplementary figures and images for: Providing Brief Personalized Therapies for Insomnia Among Workers Using a Sleep Prompt App: Randomized Controlled Trial
Source: J Med Internet Res. 2022 Jul 25;24(7):e36862. doi: 10.2196/36862 (PMC9361141; doi:10.2196/36862)

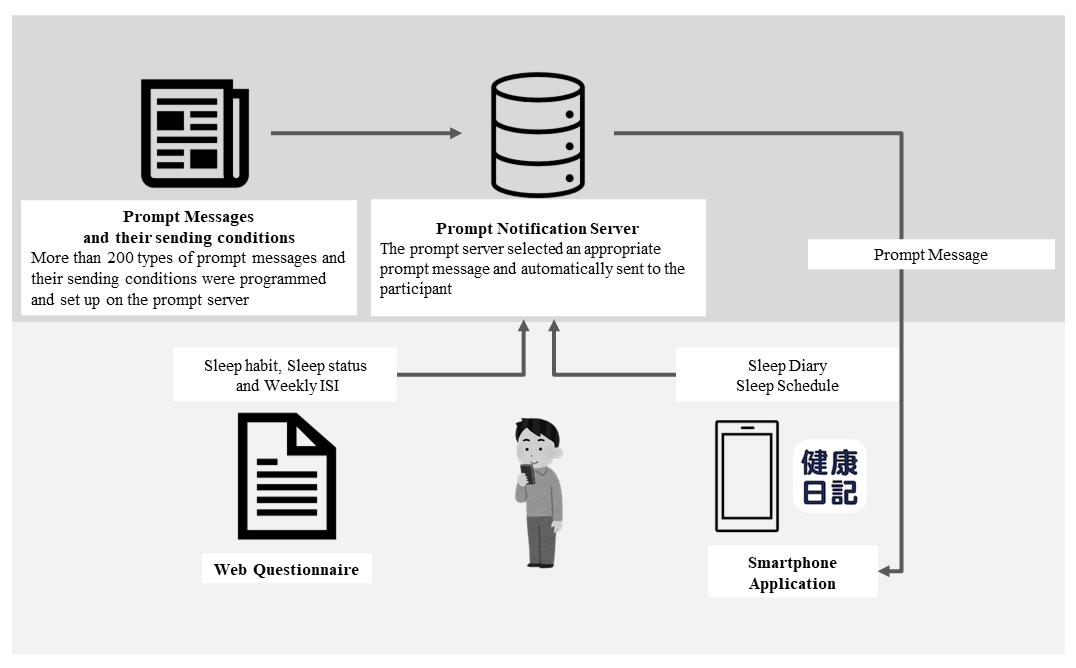

Supplement: Multimedia Appendix 1 [file jmir_v24i7e36862_app1.png]
