# Supplementary material for: Providing Brief Personalized Therapies for Insomnia Among Workers Using a Sleep Prompt App: Randomized Controlled Trial
Source: J Med Internet Res. 2022 Jul 25;24(7):e36862. doi: 10.2196/36862 (PMC9361141; doi:10.2196/36862)
Supplement: Multimedia Appendix 2 [file jmir_v24i7e36862_app2.docx]

**Multimedia Appendix (Table). The effect of SPA on sleep parameters in the sleep diary**

|  | Group | 1st week | | 2nd week | | 3rd week | | 4th week | |
| --- | --- | --- | --- | --- | --- | --- | --- | --- | --- |
|  |  | M | SD | M | SD | M | SD | M | SD |
| Sleep diary measures |  |  |  |  |  |  |  |  |  |
| Sleep Efficiency, % | Total population | 92.0 | 4.5 | 92.6 | 4.8 | 93.1 | 4.6 | 93.5 | 4.4 |
|  | ISI-8-insomniacs | 90.9 | 5.0 | 91.9 | 5.2 | 92.5 | 5.2 | 93.1 | 4.8 |
| Time in Bed, min | Total population | 451.5 | 59.9 | 461.7 | 58.7 | 460.0 | 55.7 | 466.4 | 57.1 |
|  | ISI-8-insomniacs | 460.3 | 64.2 | 465.1 | 61.1 | 469.6 | 55.0 | 470.4 | 52.8 |
| Total sleep time, min | Total population | 413.9 | 48.9 | 426.4 | 48.5 | 427.6 | 50.1 | 434.7 | 48.6 |
|  | ISI-8-insomniacs | 416.5 | 50.9 | 425.8 | 50.6 | 433.1 | 50.5 | 436.6 | 44.0 |
| Sleep Onset Latency, min | Total population | 19.1 | 14.0 | 17.8 | 17.6 | 14.9 | 14.9 | 14.6 | 15.5 |
|  | ISI-8-insomniacs | 22.0 | 14.9 | 19.4 | 19.4 | 16.8 | 16.7 | 16.0 | 16.8 |
| Wake Time After Sleep Onset, min | Total population | 12.7 | 13.6 | 14.1 | 16.0 | 13.1 | 18.5 | 13.4 | 14.1 |
|  | ISI-8-insomniacs | 16.7 | 15.5 | 19.4 | 19.4 | 15.6 | 22.6 | 16.4 | 15.9 |
| Bed out latency, min | Total population | 18.6 | 17.6 | 17.5 | 15.0 | 17.6 | 15.0 | 17.1 | 14.6 |
|  | ISI-8-insomniacs | 22.0 | 14.9 | 19.9 | 17.6 | 19.8 | 17.1 | 17.8 | 16.5 |
| Waking Up Time | Total population | 7:09 | 1:00 | 7:17 | 0:57 | 7:19 | 0:56 | 7:17 | 0:56 |
|  | ISI-8-insomniacs | 7:17 | 1:01 | 7:19 | 1:01 | 7:26 | 1:00 | 7:20 | 0:58 |
| Bedtime | Total population | 23:56 | 1:07 | 23:53 | 1:06 | 23:56 | 1:04 | 23:48 | 1:05 |
|  | ISI-8-insomniacs | 23:58 | 1:08 | 23:54 | 1:13 | 23:56 | 1:06 | 23:48 | 1:08 |

SPA: sleep prompt mobile application, SD: standard deviation.

Two participants who didn't report the sleep diary more than 4 times per week were excluded.
